# Perspective for Fibre-Hybrid Composites in Wind Energy Applications

**DOI:** 10.3390/ma10111281

**Published:** 2017-11-08

**Authors:** Yentl Swolfs

**Affiliations:** Department of Materials Engineering, KU Leuven, Kasteelpark Arenberg 44 bus 2450, 3001 Leuven, Belgium; yentl.swolfs@kuleuven.be; Tel.: +32-1637-3616

**Keywords:** fibre-hybrid composites, wind turbine blades, hybrid effects, flexure, fatigue

## Abstract

Increasing the efficiency of wind turbines will be vital for the wind energy sector to continue growing. The drive for increased efficiency is pushing turbine manufacturers to shift from glass fibre composite blades towards carbon/glass fibre-hybrid composite blades. This shift brings significant challenges in terms of optimising the design and understanding the failure of these new blade materials. This review therefore surveys the literature on fibre-hybrid composites, with an emphasis on aspects that are relevant for turbine blade materials. The literature on tensile, flexural, compressive, and fatigue performance is critically assessed and areas for future research are identified. Numerical simulations of fibre-hybrid composites have reached a reasonable maturity for tensile failure, but significant progress is required for flexural, compressive, and fatigue failure. Fatigue failure of fibre-hybrid composites in particular, requires more careful attention from both a modelling and experimental point of view.

## 1. Introduction

Climate change is an essential challenge for our society, as it causes sea levels to rise, more extreme climate events, and droughts. In December 2015, world leaders therefore signed the Paris Agreement, aiming to reduce the temperature increase to less than 2 °C above pre-industrial levels [1]. To reach such ambitious goals, countries all over the world will need to strongly reduce greenhouse gas emissions. While there are many ways to achieve this, a particularly relevant strategy is switching towards renewable energy. While many technologies are being developed and are already in use, the final solution will most likely include a mixture of different renewable energy technologies [2,3]. Wind energy is particularly interesting technology, as its price level can compete with traditional energy sources [4]. Even though wind turbines have already been installed all over the world, they still face significant technical and scientific challenges for increasing efficiency and hence lowering the cost of energy production [5,6].

The efficiency of wind energy (and other energy sources) can be split up into two aspects [5,7]. The first aspect is efficiency while in operation: how much of the wind energy is converted into electrical energy and at what cost level? The second aspect is related to lifetime and durability: what type of repair does the turbine require and what is the expected lifetime? Implementation of new materials requires improvements on both fronts.

**Figure 1:**
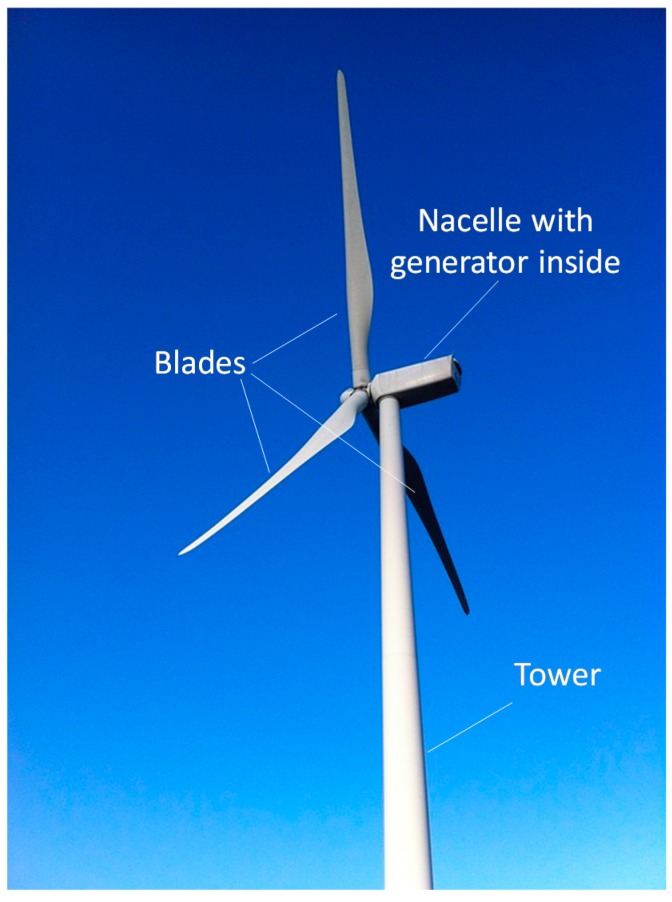
A typical horizontal-axis wind turbine.

The wind energy sector is one of the largest users of composite materials. Composites are primarily found in two components: the nacelle and the blades (see Figure 1) [8]. The nacelle is the cover housing that provides weather protection to the generator, drive train, and gearbox. The nacelle is typically made of glass fibre composites, with the main requirements being strength, corrosion resistance, and low weight [8]. The blades are arguably the most important component of a wind turbine, as the blades (1) often limit the lifetime and performance of the turbine [9], and (2) are typically the most expensive component of the turbine [9,10].

**Figure 2:**
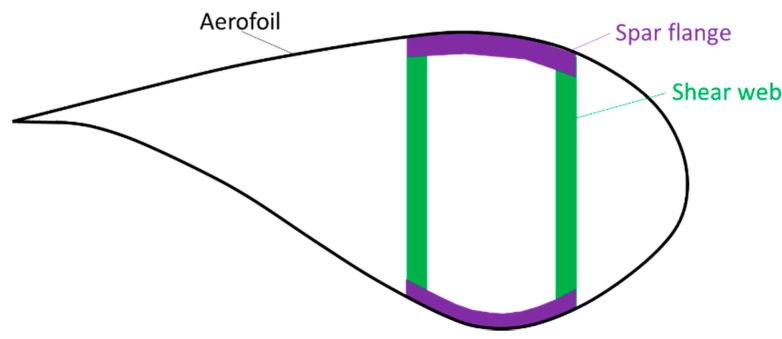
Schematic cross-section of a wind turbine blade, showing the aerofoil, shear webs and spar flanges. Inspiration for this figure comes from [7,11,12,15,16], although other designs also exist [11].

An important method to increase the operational wind turbine efficiency is to increase the blade length [11,12,13,14,15]. This is even more important for off-shore turbines, which tend to be larger than on-shore turbines [16,17,18]. Most wind turbine blades consist of a spar flange and shear webs that are covered by an aerofoil (see Figure 2), all of which are typically made of glass fibre composites [10]. As early as 2000, suggestions started appearing that the wind energy industry should switch to all-carbon fibre composites [15,19,20]. Doing so would strongly reduce the weight and blade tip deflection, while improving the fatigue resistance [8,10,12,18,21]. The lighter blade also has secondary effects, enabling lighter and cheaper components in the rest of the turbine [10]. Carbon fibre composites would also enable passive actuation: their higher anisotropy in comparison with glass fibre composites enables them to further optimise aerodynamic performance through bend-twist coupling [12,22]. Nevertheless, carbon fibre composites strongly increase the blade cost, which prohibits the use of an all-carbon fibre composite.

Even though other hybrid combinations have been suggested [23], carbon/glass fibre-hybrid composites is the solution that is starting to be adopted by turbine manufacturers. By adding carbon fibre in the regions where it is needed the most, and glass fibres in all of the other regions, a good balance between cost and performance can be achieved. The carbon fibres can, for example, be added in the spar flange [11] (see Figure 2), where they significantly increase the stiffness for a given weight or reduce the weight for a given stiffness. Adding them in the shear webs is less beneficial, as the required mechanical properties are lower in that region. It is challenging to get information on the hybridization configurations that are commonly used, as manufacturers tend not to publicise such details. It is therefore difficult to assess whether interlayer, intralayer or intrayarn configurations are common. Knowing, however, that cost is an important driver, it is highly unlikely that intrayarn configurations are used. Examples can be found in the literature on interlayer hybrids for wind turbine blades [17,24]. This configuration is typically the most cost-efficient and it is also has the fewest consequences on the manufacturing chain. Intralayer can also be cost-efficient, depending on the manufacturing technique. For pultruded spars for example, swapping a part of the glass fibre bobbins for carbon fibre bobbins is an easy-to-implement and cost-effective strategy for achieving intralayer hybrids.

The challenge in introducing fibre-hybrid composites in such large structures is related to the robustness of the overall design [7,13]. Failure of composite materials is a gradual process involving many different damage mechanisms that can interact with each other. For fibre-hybrid composites, this damage development becomes even more complicated than for non-hybrid composites. Since we expect the wind energy industry to become a key industry to use fibre-hybrid composites, it is relevant and timely to describe the current state-of-the-art in this field and identify which areas need further investigation. 

Another vital issue for wind turbine blades is non-destructive inspection and structural health monitoring [7,11,12]. This is an active research field, as it can contribute to prolonging the lifetime of wind turbines and therefore the overall energy cost. By equipping the blades with sensors, the aim is to limit maintenance costs [7]. This is particularly important for offshore wind turbines, which are more difficult to access. The use of carbon/glass hybrid composites can, in some cases, hamper or complicate a proper non-destructive inspection. Ultrasonic waves, for example, can reflect at carbon/glass interfaces, which therefore appear as damage in ultrasonic scans. Eihusen and Peters [25], for example, reported an unexplained anomaly in their measurements of the thermal conductivity of carbon/glass hybrid composites. Despite its importance, the information in the literature on non-destructive testing of fibre-hybrid composites is too limited to report on it here. More work is needed to validate the existing non-destructive testing techniques for fibre-hybrid composites.

This paper reviews both the experimental and the modelling results. Experimental campaigns tend to be time-consuming and expensive, which is often an issue for the industry. The conclusions of such campaigns are also often difficult to extend to other material combinations or loading scenarios. There is hence a global trend to move towards virtual experiments and hence to develop suitable simulation tools [26]. Such tools will contribute to improving the operational efficiency, the reliability, and the lifetime of wind turbine blades [7]. Improving and better understanding those simulation tools, combined with sufficient experimental validation, is vital to lowering the cost of wind energy and increasing its adoption worldwide.

This review paper starts by describing synergetic effects in general, as they are an important feature of fibre-hybrid composites. Next, the mechanical properties that are most important to wind turbine materials are described: tensile, flexural, compressive, and fatigue failure. Delaminations or interlaminar failure is relevant to all of these failure modes, and is relevant to failure of wind turbines in general [7,27]. It is not a failure mode, but rather a damage mechanism that contributes to these four failure modes. There is hence no separate section on this topic. Furthermore, to the best of the author’s knowledge, there is no evidence to suggest that delaminations behave differently in fibre-hybrid composites than in conventional composites.

## 2. Synergetic Effects

**Figure 3:**
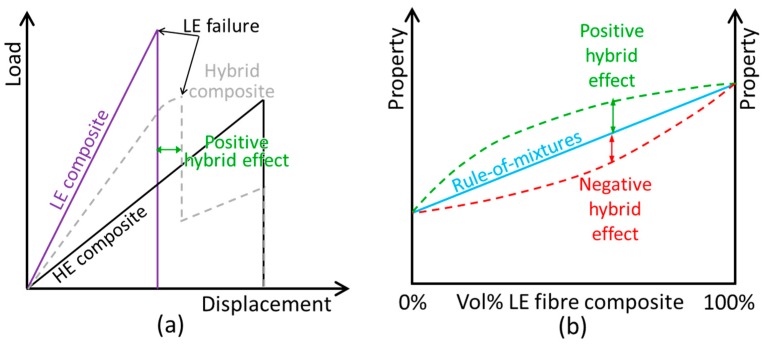
Illustration of potential definitions of the hybrid effect: (**a**) for the apparent failure strain increase of the low elongation (LE) fibres and (**b**) for a more general case (adapted from [28], with permission from Elsevier).

When combining two components, synergetic effects can arise. They appear in many different mechanical properties and materials. As a general definition, synergetic effects can be defined either as properties that are better than expected or the occurrence of behaviour that is not present in either of the constituent materials [28]. Synergetic effects are particularly relevant for fibre-hybrid composites, as large, positive synergies have been found by many authors. These are often called “hybrid effects”, and examples include:

- The initial failure strain of carbon fibre layers or bundles can be increased by up to 40% when hybridised with glass fibres (see Figure 3) [28,29].
- Even though carbon and glass fibre composites are quasi-brittle materials, they can show pseudoductility when they are hybridised in an intelligent way [30,31].
- The tensile strength of carbon/glass fibre-hybrid composites can be about 25% higher than expected based on the bilinear rule of mixtures [32].

Care should, however, be taken that the right rule-of-mixtures is chosen. For penetration impact resistance, for example, a linear rule-of-mixtures is appropriate [33], whereas a bi-linear rule-of-mixtures is more appropriate for tensile strength (see Figure 3b) [32].

A potential pitfall of measuring hybrid effects is that it relies on an accurate reference value. A good illustration is the hybrid effect for the initial failure strain in unidirectional carbon/glass composites. The required reference failure strain in this case is that of the unidirectional carbon fibre composite. Their failure strain is particularly challenging to determine, as unidirectional composites:

- Are sensitive to stress concentrations at the grips [34].
- Tend to split along the fibre direction [35], which is due to a combination of preventing the Poisson contraction within the grips, and their inherent low resistance to splitting.
- Release a large amount of energy when they fail, making it difficult to establish whether failure occurred in/near the grips or away from the grips [35].

When sandwiching the carbon fibre plies in between glass fibre plies, however, the glass fibre plies alleviate all three issues:

- They lower the stress concentrations in the carbon fibre plies [31,34].
- They hinder the splitting mechanism, as the glass fibre plies help to keep the composite together.
- They can absorb part of the energy, and simplify determining the location of the first carbon fibre ply failure [30,31].

The in situ failure strain of the carbon fibre plies therefore seems higher, but this is mainly due to a too low value for the reference failure strain. Although this is impossible to prove in hindsight, some of the literature on this type of hybrid effect seems to have been affected by this type of measurement inaccuracies [28].

The mechanisms explaining hybrid effects depend on the property under investigation. In general, however, the following can be stated [28,36]:

- Interactions among the fibres govern the hybrid effects.
- Fibre-hybrid composites with more finely dispersed microstructures allow for more interactions among the fibres, and hence larger hybrid effects.
- Constituent materials with properties that are further apart offer a larger potential for achieving strong hybrid effects.

## 3. Tensile Failure

Tensile failure of fibre-hybrid composites has received significantly more attention than other mechanical properties. Those studies also provide a framework for understanding some of the other mechanical properties that are relevant to wind turbine blades. While pure tension is not that important for wind turbine blades, flexural loading is a common loading scenario (see Section 4). Given the importance of flexural loads in wind turbine blades, it is vital to understand how failure develops on the tensile side during flexural loading.

### 3.1. Failure Development

To understand how tensile failure develops in a fibre-hybrid composite, the longitudinal tensile failure of a unidirectional composite must first be understood. Such failure is governed by a combination of the Weibull strength distribution of the fibres and stress concentrations around fibre breaks [37]. The Weibull strength distribution controls the strength of the fibres, which implies that they all fail at a different strain and strength level. Once fibres have broken, their load is shed to the nearby fibres. These nearby fibres are therefore subjected to stress concentrations, which increase their failure probability. This creates a tendency to develop clusters of fibre breaks, which further intensify the stress concentrations. At some point, a critical cluster develops, which propagates unstably and causes final failure.

When a unidirectional composite contains not one but two fibre types, the failure development is altered in several ways:

- The same sample size in hybrid composites contains fewer fibres of the low elongation type than composites with only low elongation fibres. This implies that a size effect can contribute towards an increased failure strain.
- The high elongation fibres tend to constrain cluster size, as they are less likely to fail than the low elongation fibre type. This delays the growth of clusters of fibre breaks, and therefore increases the failure strain [28].
- The difference in coefficients of thermal expansion can cause thermal residual stresses upon cooldown after curing. This is known to cause compressive stresses in the carbon fibre plies in carbon/glass fibre-hybrid composites, which counteract the externally applied loads. This contribution is, however, relatively small in general [31,38,39,40], and even smaller for wind turbine blades, as they are typically cured either at 20–40 °C for infusion processing or 80 °C for prepreg-based technologies.
- The broken fibres release their strain energy when they break, and cause stress waves to propagate through the composite. The presence of two fibre types affects this propagation and can potentially lower the dynamic stress concentrations [28,41]. The number of studies on this effect is limited, making it difficult to assess its importance.

### 3.2. Influencing Parameters

Many parameters influence the hybrid effect for initial failure strain in longitudinal tension, and it is difficult to assess all of them experimentally. Some of the parameters are difficult, if not impossible, to change experimentally. For example, the Weibull distribution for fibre strength cannot be readily changed, apart from changing to a different fibre type altogether. Models are therefore needed in assessing which parameters control the hybrid effect. 

The first models started appearing in the seventies and eighties [39,42,43,44]. These were relatively simple and required strong assumptions, but nevertheless already captured some basic influencing parameters. In the past decade however, new and more advanced models started appearing [31,45,46,47,48,49]. These models confirmed some of the conclusions of the earlier models, but also shed new light on others.

The relative volume fraction of both fibre types is one of the key influencing parameters, as this fraction is easy to change. It also has a significant influence on the hybrid effect and is important for other mechanical properties such as stiffness. Many researchers have experimentally changed the relative volume fraction and assessed its influence. These studies concluded that a lower fraction of low elongation fibres increases the failure strain of that fibre type, and hence leads to a larger hybrid effect [28,31,36,38]. Those reports, however, are not always unanimous and consistent. The modelling support for this conclusion, however, is overwhelming and unanimous [31,45,48,49,50].

The dispersion of the fibre types is also an important parameter that is governing the hybrid effect [45]. Many authors have attempted to investigate this effect experimentally, but their results are often difficult to interpret as the relative volume fraction was changed at the same time [31,38,51]. In some cases, however, the dispersion was varied independently of the hybrid volume fraction, which led to clear results [52,53]. The initial models were not able to separate both of the effects either [39,43,54,55,56,57]. Later developments based on the global load sharing models had the same limitation [48,49]. Some of the more recent modelling results, however, can separate both effects, and their conclusions are unanimous: a better dispersion increases the hybrid effect [45,47]. An important note is that the hybrid effect seems to disappear for interlayer carbon/glass hybrids with carbon fibre layer thicknesses above 100 µm [31]. This implies that standard ply thicknesses, which are above 100 µm and are commonly used in wind turbine blades, should not lead to hybrid effects. This indicates that further improvements may be possible if wind turbine blades are made more of thinner plies or, more generally, a better dispersed hybrid. It should be noted though that finer dispersion or thinner plies tend to be more expensive.

The Weibull modulus for fibre strength also affects the magnitude of the synergies. Due to practical limitations, this has never been investigated experimentally. The first, simple models for the hybrid effect already supported this statement [39,50], but their conclusions were weakened by several points. Firstly, Zweben [39] assumed that both fibre types have the same Weibull modulus, which prevented him from discerning the relative contribution of those two Weibull moduli. Fukuda [56], on the other hand, did not consider failure of the high elongation fibre, which implies that its Weibull modulus does not appear in his equations. Later models did not have these limitations and convincingly showed that a lower Weibull modulus of the carbon fibres increases the hybrid effect [45,49]. Note that a low Weibull modulus implies a large scatter in the fibre strength. The importance is, however, limited to the Weibull modulus of the low elongation fibre. For typical fibre-hybrid composites, the Weibull modulus of the high elongation fibre did not affect the hybrid effect [49].

The effect of the failure strain ratio of the two fibre types has long been unclear. Two of the earliest models came to opposite conclusions: Zweben [39] found a very strong influence, whereas Fukuda [56] found no influence at all. This was due to the severe assumptions and simplified packings that were used in both models. Swolfs et al. [46] later showed that increasing the failure strain of the high elongation composite was beneficial, but that the effect levelled off for failure strain ratios above 2. This threshold ratio is not universal, but depends on the specific combination of fibres and matrix.

### 3.3. Size Scaling

**Figure 4:**
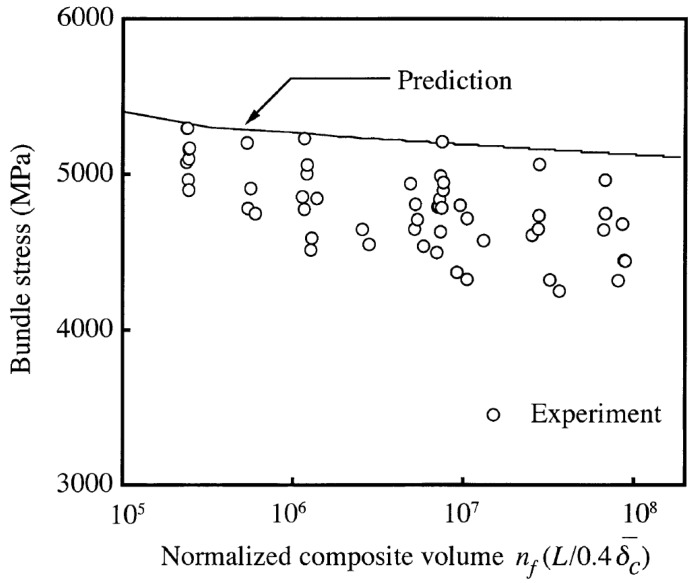
An increased composite volume leads to a decreased composite strength (reprinted from [58], with permission from Elsevier).

Wind turbine blades are one of the world’s largest composite structures, making the proper understanding of size scaling effects vital. These effects are often challenging to measure and predict, as there are practical limitations to the size of real, as well as virtual, specimens. A landmark paper in this respect was Okabe and Takeda in 2002 [58]. They performed tensile tests on unidirectional composites with the tested volume changing over 2.5 orders of magnitude. Okabe and Takeda found that the strength of the largest samples was about 10% lower than that of the smallest samples (see Figure 4). Their model predicted a similar decrease with increasing size, although that decrease was smaller than the experimentally measured one.

Size scaling is a challenging topic for micromechanical simulations. Some simulation strategies are analytic, and hence fast, such as the global load sharing scheme that was developed by Curtin [59,60] and later improved by Neumeister [61] and Hui et al. [62] or the hierarchical scaling law of Pimenta and Pinho [63]. Such analytical approaches however use significant assumptions. The global load sharing scheme is insensitive to size effects, as size is not explicitly present in the equations. The hierarchical scaling law is capable of predicting size effects, but it is challenging to assess how its assumptions and simplifications affect the size scaling effects. On the other side of the spectrum are the finite element models, which are very detailed and take into account most of the relevant micromechanisms [64,65]. Such models, however, are computationally very expensive, as they can run up to days or weeks. They are therefore limited to a small number of fibres (typically a few hundred or less). Other modelling approaches are in between these two extremes [66,67,68,69,70,71]. Choosing the right simulation tool for doing size scaling studies and understanding its benefits, drawbacks, limitations, and assumptions is therefore essential. The reader is referred to the more detailed reviews that are available in the literature [37,72], although these reviews do not directly address fibre-hybrid composites.

The literature on size scaling of the hybrid effect is scarce. The hybrid effect inherently implies a size effect, as it depends on ply thickness, or more generally, the dispersion of the fibre types. Systematic studies using the same ply thickness or dispersion, but changing the sample width or length are rare. To the best of our knowledge, there is only a single source. Jones and DiBenedetto [57] predicted that the hybrid effect increased with an increasing gauge length. Increasing the model length from 20 to 200 mm increased the hybrid effect from 25 to 30%, 51 to 59%, or 82 to 92%, depending on the specific configuration. They attributed this to another feature of fibre-hybridisation that they found: the low elongation plies in a fibre-hybrid composites tend to have a narrower strength distribution than a composite with only low elongation fibres. The consequence of this narrower distribution is that fibre-hybrid composites are less sensitive to size scaling than non-hybrid composites, which explains the increasing hybrid effect with an increased size. Further research in this area is required to support the development of wind turbine blades using fibre-hybrid composites.

Sørensen [7] notes an interesting difference between the wind energy and aerospace industry that is related to size scaling: the aerospace industry aims to reduce costs by limiting the number of tests at various length scales, whereas the wind energy industry is increasingly testing and modelling at various length scales. This includes microscale [73,74], coupon [75] and component scale testing [9], and trying to link this together via multi-scale modelling [26].

### 3.4. Multidirectional Composites

Research on tensile failure of fibre-hybrid composites has primarily focused on longitudinal tension in unidirectional composites. Models for multidirectional composites tend to ignore fibre break development, and assume either a constant/uniform strength or a certain strength distribution for the 0° plies. Such models hence fail to capture the hybrid effect, as described in Section 3.1. Theoretically however, it is well recognised that cracks in off-axis plies locally increase the stresses in the 0° plies [76]. That should hence increase the probability for fibre break development and reduce the in situ strength of the 0° plies.

Some attempts have been made to model fibre breaks in multidirectional composites. The fibre break model in Scott et al. [77] did take into account the effect of the 90° plies on the 0° plies, although they did not explain how this was done. Similarly, to the best of our knowledge, there are no models in the literature that predict fibre breaks in non-crimp fabrics, weaves, or other textile composites. At the moment, the influence of (1) off-axis cracks; (2) delaminations; (3) crimped yarns; and, (4) stitching yarns on fibre break development in 0° plies in fibre-hybrid composites remains unknown. 

### 3.5. Conclusions

The hybrid effect for the tensile failure of the low elongation plies in unidirectional composites can be significant, but they tend to be difficult to measure. The experimental evidence for certain parameters is either lacking or weak, whereas the modelling predictions seem to agree with each other in most cases. Models are, however, not perfect either, as they can be overly simple and their outcome may be biased in certain cases. Even though this field is already relatively mature, more efforts are required in experimental validation of the state-of-the-art models.

The amount of information on the overall tensile response, including what happens after the failure of the low elongation plies, is more limited and less detailed. The damage mode map approach is useful in determining the type of failure that can be expected [78]. It is, however, less relevant to wind turbine blades, as they will not or should not be loaded past the failure of the carbon fibre plies.

## 4. Flexural and Compressive Failure

**Figure 5:**
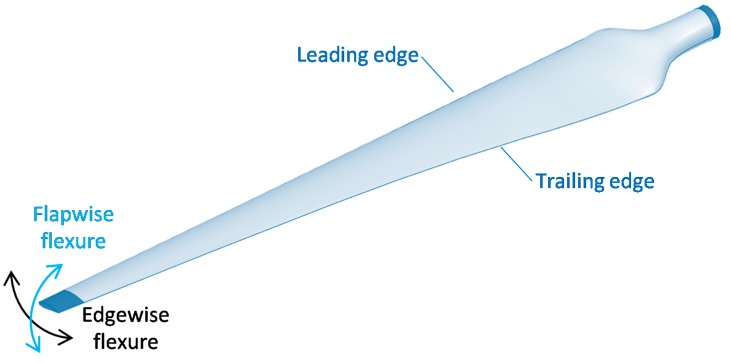
Illustration of the main flexural loads on wind turbine blades.

Flexure is an essential loading scenario for wind turbine blades, occurring both flapwise and edgewise (see Figure 5) [8,11,12]. These two types of flexure are responsible for the majority of damage that occurs in blades. Flapwise flexure is particularly important, as the loads tend to be higher and the stiffness in this direction needs to be sufficiently high to prevent collision with the tower [11,13].

In essence, flexure is a combination of tension, compression, and shear. As with pure tension, the modulus of fibre-hybrids in flexure can be predicted based on the classical laminate theory. Two notable exceptions were reported in the literature: (1) the transverse tensile modulus of continuous carbon fibre/self-reinforced composites [79] and (2) the longitudinal modulus of discontinuous fibre composites [80]. Apart from those exceptions, nearly all of the reports agree that conventional approaches for modulus predictions also apply to fibre-hybrid composites [29,40,81,82,83,84]. An important consideration, however, is that simple rule-of-mixtures only work for tension, and that flexure requires the classical laminate theory or other, more advanced, tools. 

**Figure 6:**
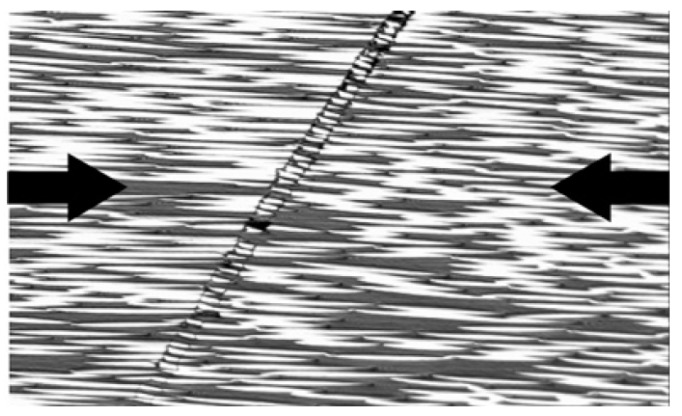
Example of kink band formation in a carbon fibre/epoxy specimen (reprinted from Laffan et al. [100], with permission from Elsevier).

A thorough study on flexural properties of fibre-hybrid composites has been performed by Dong, Davies and co-workers [85,86,87,88,89,90,91,92,93]. Their research indicated that the classical laminate theory agrees well with more advanced finite element predictions, as well as with experiments [85]. They also developed finite models for predicting the flexural strength of hybrid composites by incorporating tensile and compressive failure models [85,86,87]. Simple rules-of-mixtures were used for tensile strength, thus using a deterministic strength value. Their initial works [85,86,87] predicted compressive failure through microbuckling and kinking (see Figure 6) using the Lo-Chim model [94]. Later on, their models [92,93] were extended by including compressive failure through delamination/shear using the model of Chamis et al. [95], exploring the Budiansky-Fleck model for microbuckling and kinking [96,97], and other failure theories, such Azzi-Tsai-Hill [98] and Tsai-Wu [99].

All of the above models assumed a constant, uniform strength for the individual plies, which implies that they did not capture size scaling effects. Size scaling is more important for flexure than for tension, as the flexural stresses are more localised. Dong et al. [85] did empirically correct for this, by assuming that the bending strength is 35% higher than compression strength.

Ideally, flexural models would combine the tensile models described in Section 3 with models for compressive failure. Many authors have devised models for compressive failure, such as the maximum stress criterion, Tsai and Wu [99], Lo and Chim [94], Puck [101], Pinho et al. (LaRC05) [102], and Budiansky et al. [96,97]. Some criteria, such as maximum stress and Tsai-Wu, are curve-fitting approaches, whereas others are more physically based [97,101,102]. Nevertheless, several authors have stated that the compressive failure models are relatively immature when compared to tensile failure models [97,103,104,105]. The reasons stated are:

- Experimental determination of compressive failure envelopes is particularly difficult due the free edge effects and sensitivity to material defects and imperfections in the test setup [103,104].
- Compressive failure is highly sensitive to fibre misalignment, which is difficult to control or prevent [97,104,105].
- Compressive failure predictions require a reliable description of matrix plasticity in a complex stress state [97,103,104].

To the best of our knowledge, only one model has been developed for compressive failure of fibre-hybrid composites. Mishnaevsky and Dai [47] predicted that adding carbon fibres to glass fibre composites can reduce the compressive strength, which is explained by the lower compressive strength of the carbon fibre composites. This is in line with the review of Kretsis [36], which reported more negative than positive synergies for compressive strength. This may turn out to be the key obstacle for the introduction of carbon/glass hybrid composites in wind turbine blades.

## 5. Fatigue Failure

Fatigue failure is the tendency of a material to fracture under repeated loading below the static strength. The failure occurs due progressive damage development at the microscale, such as growth of delaminations or fibre-matrix debonds, and fatigue of the constituent materials [106]. Fatigue can occur in different loading conditions, such as tension, flexure and compression, or even combined loading conditions. These conditions are often captured in the parameter R, which is the ratio of maximum load over minimum load. The loads in coupon tests often vary sinusoidally with constant amplitude. Real load conditions in wind turbines, however, are much more complex [12]. 

According to many authors, the fatigue performance has become the design driver for wind turbine blades [12,16,18,20,107,108]. The occurrence of fatigue is mainly linked to the edgewise and flapwise flexural loading, as shown in Figure 5 and described in Section 4. The loading modes are mostly tension-tension (e.g., R = 0.1) and compression-compression (e.g., R = 10), although tension-compression (e.g., R = −1) can also occur in some regions of the blade [12,109].

Prior to causing earlier failure, repeated loading has an important consequence: it causes a stiffness degradation [109]. This is important to capture, as too big a loss in stiffness can cause the blade to collide with the tower [110]. The degradation is the result of the development of cracks in off-axis plies and delaminations and has received extensive attention in the literature for non-hybrid composites [110,111]. This has received significantly less attention in the literature on fibre-hybrid composites. Our hypothesis is that much of the fibre-hybrid work focused on longitudinal loading of unidirectional composites [107,112,113,114], which do not or hardly suffer from stiffness degradation [115,116].

Fatigue failure of composites is challenging to analyse, as many damage mechanisms interact with each other. This is not only true for multidirectional and textile composites, but also for unidirectional composites [115,117]. Many models in the literature rely on extensive experimental campaigns, and are essentially advanced curve-fitting approaches. They typically offer little insight into the mechanisms that are controlling fatigue damage development. Mechanistic models, on the other hand, aim to capture the actual fatigue damage mechanisms. They are more challenging to develop, but they also offer a greater potential for understanding the mechanisms. The tendency in the literature is shifting towards mechanistic models [111,117].

**Figure 7:**
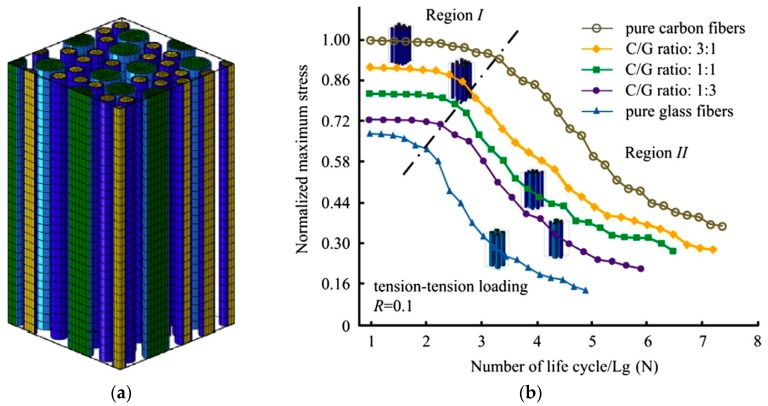
The fatigue model of Dai and Mishnaevsky for unidirectional fibre-hybrid composites: (**a**) 3D view of the representative volume element and (**b**) predicted S-N curves for carbon/glass hybrid composites (reprinted from [107] with permission from Elsevier).

To thoroughly understand the influence of fibre-hybridisation on fatigue, models specifically for fibre-hybrid composites are required. To the best of our knowledge, the model of Dai and Mishnaevsky [107] is the only such model that is currently available in the literature. This three-dimensional finite element model (see Figure 7a) captures fatigue damage development through a Paris law in combination with the extended finite element method. The size of their unit cell was 42 µm × 42 µm × 125 µm, which implies that only a few tens of fibres were modelled (see Figure 7a). Dai and Mishnaevsky [107] concluded that a higher fraction of carbon fibre increased the fatigue life of carbon/glass fibre-hybrid composites in tension-tension fatigue (see Figure 7b). In compression-compression however, the trend was the opposite: a higher fraction of carbon fibre deteriorated the fatigue life. This difference was explained by the fact that carbon fibres are more prone to kinking failure than glass fibres.

Several authors have experimentally investigated the fatigue performance of fibre-hybrid composites [18,32,52,113,118,119,120,121]. Most authors reported an increased fatigue life upon the addition of carbon fibres to a glass fibre composite [18,32,52,113,118,119], whereas some studies were more difficult to interpret [120,121]. It should be noted that the majority of these studies performed tension-tension fatigue. Bach [121] also found significant improvements in tension-tension fatigue by adding carbon fibres to a glass fibre composites, but found disappointing results in tension-compression. This matches well with the modelling results of Dai and Mishnaevsky [107], who attributed this to the poor compressive performance of carbon fibre composites. 

Finally, several other effects are noteworthy:

- The fatigue performance is known to be sensitive to the matrix and interface [106,117]. Choosing the right matrix may be more challenging for fibre-hybrid composites, as the optimal matrix for one fibre type may be suboptimal for the other type.
- The addition of 90° layers makes composites more prone to fatigue damage development [18], as the 90° cracks often trigger failure in the 0° plies [122].
- The fatigue life also depends on the textile architecture [108,123], which is likely to be modified when fibre-hybridisation is applied.
- The fatigue life of glass fibre composites is sensitive to moisture, and hybridising them with carbon fibre composites tends to reduce that sensitivity [32,118,119].

Much more extensive work is needed before these effects are not only understood, but are also reliably predicted by models. 

## 6. Conclusions

Fibre-hybrid composites have great potential for the use in wind turbine blades, and most manufacturers are either already using them or are considering to do so. This provides a large market potential in the near future.

In wind turbine blades, tension is not the only relevant loading scenario. Flexure and compression are also relevant loading scenarios. The most important loading scenario is fatigue due to flexure of the blades. Advancing the use of fibre-hybrid composites in the wind energy industry will require more detailed experimental and numerical studies of all those properties, but for fatigue in particular.

Numerical simulations for tensile failure of fibre-hybrid composites are relatively advanced, and the overall failure development is reasonably well understood. This is however not the case for flexural, compressive, and fatigue failure of fibre-hybrid composites. These types of failure behaviour, in particular, require more dedicated efforts to better understand them and to provide the industry with validated tools than can be used in the turbine blade design process. It also remains challenging to model the entire problem of large, curved structures with complex layups, loading conditions, and a wide range of manufacturing defects. Combining models and experiments on microscale, coupon scale and full component scale currently remains the most suitable approach. Given the size of wind turbine blades, multi-scale modelling will be vital to link the different scales together.

Several works cited in the introduction claimed that adding carbon fibre composites would improve the fatigue performance when compared to all-glass fibre composites [8,10,12,18,21]. This notion is based on the fact that it is generally believed that carbon fibre composites are less sensitive to fatigue than glass fibre composites. This notion is, however, based on fatigue data in the literature that is predominantly based on tension-tension fatigue mode. The evidence in Section 5 supports this notion for tension-tension fatigue, but raises an important concern for tension-compression and compression-compression modes. Those modes require more thorough investigations, as they may prove to be the stumbling block for introducing carbon/glass hybrids in wind turbine blades.
